# Improvement of severe myalgic encephalomyelitis/chronic fatigue syndrome symptoms following surgical treatment of cervical spinal stenosis

**DOI:** 10.1186/s12967-018-1397-7

**Published:** 2018-02-02

**Authors:** Peter C. Rowe, Colleen L. Marden, Scott Heinlein, Charles C. Edwards

**Affiliations:** 1 Division of General Pediatrics and Adolescent Medicine, Department of Pediatrics, Johns Hopkins University School of Medicine, 200 N. Wolfe St., Room 2077, Baltimore, MD 21287 USA; 2 Lifestrength Physical Therapy, Inc, 110 West Road, Suite 105, Towson, MD 21204 USA; 3 Maryland Spine Center, Mercy Medical Center, 301 St. Paul Place, Baltimore, MD 21201 USA

**Keywords:** Cervical stenosis, Cervical myelopathy, Chronic fatigue syndrome, Myalgic encephalomyelitis, Postural tachycardia syndrome, Orthostatic intolerance

## Abstract

**Background:**

Myalgic encephalomyelitis/chronic fatigue syndrome (ME/CFS) is a potentially disabling disorder. Little is known about the contributors to severe forms of the illness. We describe three consecutive patients with severe ME/CFS whose symptoms improved after recognition and surgical management of their cervical spinal stenosis.

**Methods:**

All patients satisfied clinical criteria for ME/CFS and orthostatic intolerance, and were later found to have cervical spinal stenosis. Overall function was assessed before and after surgery using the Karnofsky score and the SF-36 physical function subscale score.

**Results:**

Neurological findings included > 3+ deep tendon reflexes in 2 of 3, a positive Hoffman sign in 2 of 3, tremor in 2 of 3, and absent gag reflex in 1 of 3. The cervical spine canal diameter in the three patients ranged from 6 to 8.5 mm. One had congenital cervical stenosis with superimposed spondylosis, and two had single- or two-level spondylosis. Anterior cervical disc replacement surgery in two patients and a hybrid anterior cervical disc fusion and disc replacement in the third was associated with a marked improvement in myelopathic symptoms, resolution of lightheadedness and hemodynamic dysfunction, improvement in activity levels, and improvement in global ME/CFS symptoms.

**Conclusions:**

The prompt post-surgical restoration of more normal function suggests that cervical spine stenosis contributed to the pathogenesis of refractory ME/CFS and orthostatic symptoms. The improvements following surgery emphasize the importance of a careful search for myelopathic examination findings in those with ME/CFS, especially when individuals with severe impairment are not responding to treatment.

## Background

Myalgic encephalomyelitis/chronic fatigue syndrome (ME/CFS) is a relatively common, complex, multisystem disorder that is associated with a substantial impairment in pre-illness levels of activity and quality of life. [1, 2] Many affected individuals are disabled. [3, 4] Daily function in adults is comparable to patients with congestive heart failure or multiple sclerosis [2–4], underscoring the need for better understanding of the pathophysiology and more effective treatments. Current treatment of ME/CFS is generally symptomatic and supportive [5, 6], using medications directed at specific symptoms such as headaches, insomnia, and pain, and at co-morbid problems such as orthostatic intolerance. Little is known about the risk factors, optimal treatment, and prognosis for severe cases.

Cervical myelopathy refers to dysfunction of the spinal cord, and can present with a myriad of subtle symptoms ranging from limb paresthesias, difficulties with manual dexterity, and imbalance [7–9] occasionally in the absence of neck pain [10]. Cord compression caused by bulging cervical discs (spondylosis) is the most common cause of cervical myelopathy, and is more likely to occur when the canal is congenitally narrowed. A sagittal antero-posterior (AP) diameter of the cervical canal of less than 13 mm is a strong risk factor for the development of myelopathy [11]. Other factors that can reduce the functional diameter of the spinal canal can include the presence of osteophytes and thickening of the ligamentum flavum or posterior longitudinal ligament—all of which are more common in older adults—as well as narrowing imposed by dynamic movement. Using flexion and extension MRI imaging in individuals with cervical spondylosis, Muhle and colleagues have shown that cervical spinal stenosis increases in both flexion and extension positions [12], consistent with earlier cadaver studies [13]. Spondylotic narrowing also results in adverse mechanical tension and deformative strain within the cord, which has been proposed as another mechanism of neurological symptom provocation and injury [14, 15].

We report three young adult women who had disabling impairments in daily function that were unresponsive to medical management of their ME/CFS and orthostatic intolerance. All experienced substantial global improvements after clinical recognition and surgical management of cervical spinal canal stenosis and myelopathy.

## Methods

### Participants

This retrospective case series describes three consecutive patients referred to the Johns Hopkins Chronic Fatigue Clinic who (1) satisfied the Fukuda criteria for CFS [16] as well as both the Canadian Consensus Criteria [17] and the Institute of Medicine criteria for ME/CFS [1], (2) had evidence of daily orthostatic intolerance symptoms associated with hemodynamic evidence of either neurally mediated syncope or postural tachycardia syndrome (POTS), (3) were unable to work or attend school, (4) had evidence of cervical stenosis (as described below), and (5) were minimally responsive to medical, psychiatric, and physical therapy management over a period of at least 5 years.

All individuals were evaluated by a generalist physician (PCR) who performed a physical examination that included a Beighton score of joint hypermobility (range 0–9, with higher scores indicating increased joint laxity) [18], neurodynamic range of motion maneuvers, and a neurological examination. Deep tendon reflexes were graded 0–4. The Hoffman sign was performed with the patient seated and with the head and neck in a neutral position. With the patient’s distal interphalangeal joint of the middle finger supported by the examiner’s index finger, the examiner’s thumb made an abrupt downward flicking of the patient’s distal phalanx. The Hoffman sign was considered positive if there was flexion of the patient’s ipsilateral thumb or index finger [19].

### Imaging

All patients underwent cervical spine magnetic resonance imaging (MRI) in a closed 1.5T MRI imaging system. Measurements of the sagittal spinal canal diameter were described by the reporting radiologist.

The clivo-axial angle was the angle formed between the posterior clivus and the posterior axial line. A clivo-axial angle of 150–165° is considered normal [20] and angles less than 135° are considered pathological [21].

### Surgical technique

All patients were evaluated by the same spine surgeon (CCE). Through a 1-inch transverse incision in a skin crease to the left of midline on the anterior neck, using standard techniques under microscopic visualization, the disc annulus, nucleus pulposus, endplate cartilage, and posterior longitudinal ligament were removed with a high-speed burr and manual curettes. Posterior projecting endplate osteophytes were thinned with a burr and removed with curettes. All soft-tissues (disc herniation and fibrocartilage) projecting dorsal to the posterior vertebral endplate were removed to provide for thorough spinal canal expansion and spinal cord decompression. The posterior portions of uncovertebral joints were thinned and removed to provide generous space for the exiting nerve roots.

After satisfactory expansion of the spinal canal and foramen, reconstruction was achieved with placement of a mechanical artificial disc implant. Measurements and trials determined the optimal implant size. A groove was fashioned in the central portion of the endplates using a reamer to accommodate the bi-convex shape of the implant. Under fluoroscopic guidance, the implant (Prestige Disc or Bryan Disc, Medtronic) was inserted. For patient 2, two discs were pathologic. Decompression was performed at both levels as described, and a Bryan Disc implant positioned at C5–6. Reconstruction at C6–7 involved a fusion of C6 to C7. A plastic support (Cervical Cage, Calvary Spine) filled with calcium phosphate crystals (Vitoss, Stryker) and bone marrow aspirate was placed in the interbody space. A titanium plate (Atlantis, Medtronic) was affixed to the anterior cortex of C6 and C7 with screws to provide stability to the segment.

### Outcome measurements

Improvements in health-related quality of life before and after cervical disc replacement surgery were assessed using (1) a clinician-assigned Karnofsky score (range 0–100) [22], and (2) the self-reported Medical Outcomes Study 36-item short-form health survey (SF-36) physical function (PF) subscale score (range 10–30) [23]. Higher scores indicate better function on both measures. Changes in heart rate during orthostatic stress were measured before and after surgery using a standing test. The standing test involved 5 min of supine posture, then 10 min of standing, followed by another 2 min supine. When standing, the individual leaned against the office wall, with feet 2–6 inches away from the wall. Movement and discussion were discouraged during the period upright. POTS was diagnosed if the difference between the lowest supine heart rate (HR) and the peak HR during standing was ≥ 30 beats per minute (bpm) for those > 19 years and ≥ 40 bpm for those tested when 19 years of age or less [24]. The study was approved by the Institutional Review Board of the Johns Hopkins Medical Institutes. Informed consent was waived for a retrospective study using data collected as part of routine care.

## Results

### Case reports

*Patient 1* was well until a viral gastrointestinal illness at age 12. Thereafter she reported progressive fatigue along with unrefreshing sleep, post-exertional malaise (PEM), problems with short-term memory and attention, headache, myalgias, arthralgias, sore throat, and tender glands, thus satisfying the clinical criteria for ME/CFS. In addition, she had daily lightheadedness and frequent anxiety. At age 15, her supine heart rate was 86 beats per minute (bpm), rising 64 bpm to a peak of 150 bpm after 3 min of standing, associated with increased fatigue, headache, lightheadedness, and dyspnea, consistent with POTS. The physical examination at 15 years revealed a head-forward posture, as well as abnormal responses bilaterally on the upper limb neurodynamic test with a median nerve bias (a measure of neural tension) [25]. Her physical therapist (SH) noted tenderness and increased resting tone in the mid-cervical muscles on the right side. The Beighton score was 0.

**Fig. 1:**
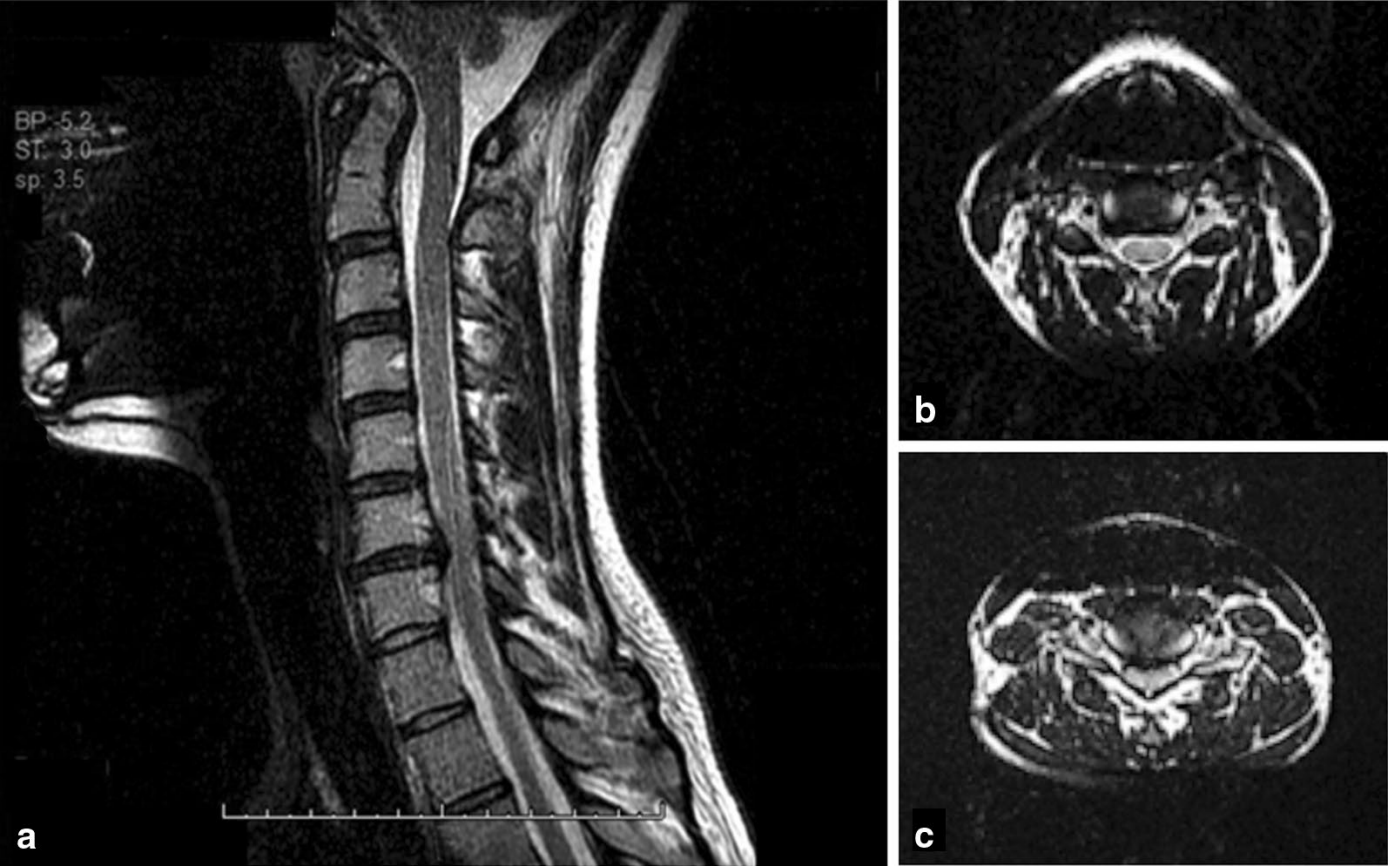
Cervical spine MRI images (Patient 1). **a** The sagittal cervical cord MRI image showed a clivo-axial angle of 118°, as well as a broad right paracentral disc bulge at C6–7 causing mild cord compression and canal stenosis, with an antero-posterior (AP) cervical canal diameter of 6 mm at that level. The cervical canal diameter from C3 to C7 was congenitally narrow at 8 mm. **b** A transverse image at C5–6. **c** A transverse image at C6–7 illustrating the site of cord compression

She was unable to attend her last 2.5 years of high school due to the severity of her symptoms. She was minimally responsive to medications directed at POTS and anxiety. The neurological examination was initially normal, but when repeated at age 19 due to emergence of tinnitus, she had developed a positive right Hoffman sign. Her mother had undergone surgical decompressions at ages 34 and 43 years for congenital cervical spinal stenosis. Patient 1′s cervical spine MRI showed a congenitally narrow cervical spinal canal, with spondylotic stenosis and an AP diameter of 6 mm at C6–7 (Fig. 1).

At age 21, she underwent cervical disc replacement at C6–7. She reported some improvement in neck discomfort, tachycardia, and cognitive fogginess in the first week after surgery. Two months after surgery, repeat physical therapy evaluation showed a normal upper limb neurodynamic test with a median nerve bias, and resolution of the neck muscle tightness. She began part-time employment. Exercise tolerance increased gradually, and her lightheadedness, tachycardia, and anxiety decreased significantly in frequency and intensity. By 6 months post-operatively, she was able to work 12-h shifts as a horse wrangler, which involved saddling and feeding horses, leading trail rides, and cleaning barn stalls. One year after surgery she began full-time university studies as well as up to 20 h of work each week. At 5 years of follow-up, she has no further ME/CFS symptoms, and has continued to enjoy full activity with no restrictions.

*Patient 2* had been generally healthy and active, employed full-time until age 29 when she initially developed profound fatigue, unrefreshing sleep, PEM, difficulties with short-term memory and concentration, headaches, myalgias, and arthralgias, thus meeting clinical criteria for ME/CFS. Neurological symptoms included burning in the legs with standing, numbness in the limbs, electric shock sensations in the arms, difficulty swallowing, and clumsy gait. She developed anxiety and depression as well. The patient had several features of hereditary connective tissue disorders, including a pectus excavatum repair at age 12 years and leg varicosities treated with vein ligation and stripping at age 28, but did not have generalized joint hypermobility (Beighton score was 2). Her sister had scoliosis and Chiari I malformation.

At age 30, deep tendon reflexes (DTRs) were rated 2–3+. Electroencephalogram, electromyogram, and nerve conduction velocity studies were normal. An MRI of the brain and complete spine revealed degenerative disc desiccation extending from C3 through C7, rated as mild. The consulting neurologist felt it was very unlikely that the disc protrusions were contributing to her symptoms. At age 31, a 70-degree head-up tilt table test showed a 61 bpm increase in HR from 65 bpm supine to a peak of 126 bpm at 10 min, with reproduction of her typical orthostatic symptoms, but without orthostatic hypotension, thus consistent with POTS. Medications directed at her POTS were ineffective.

**Fig. 2:**
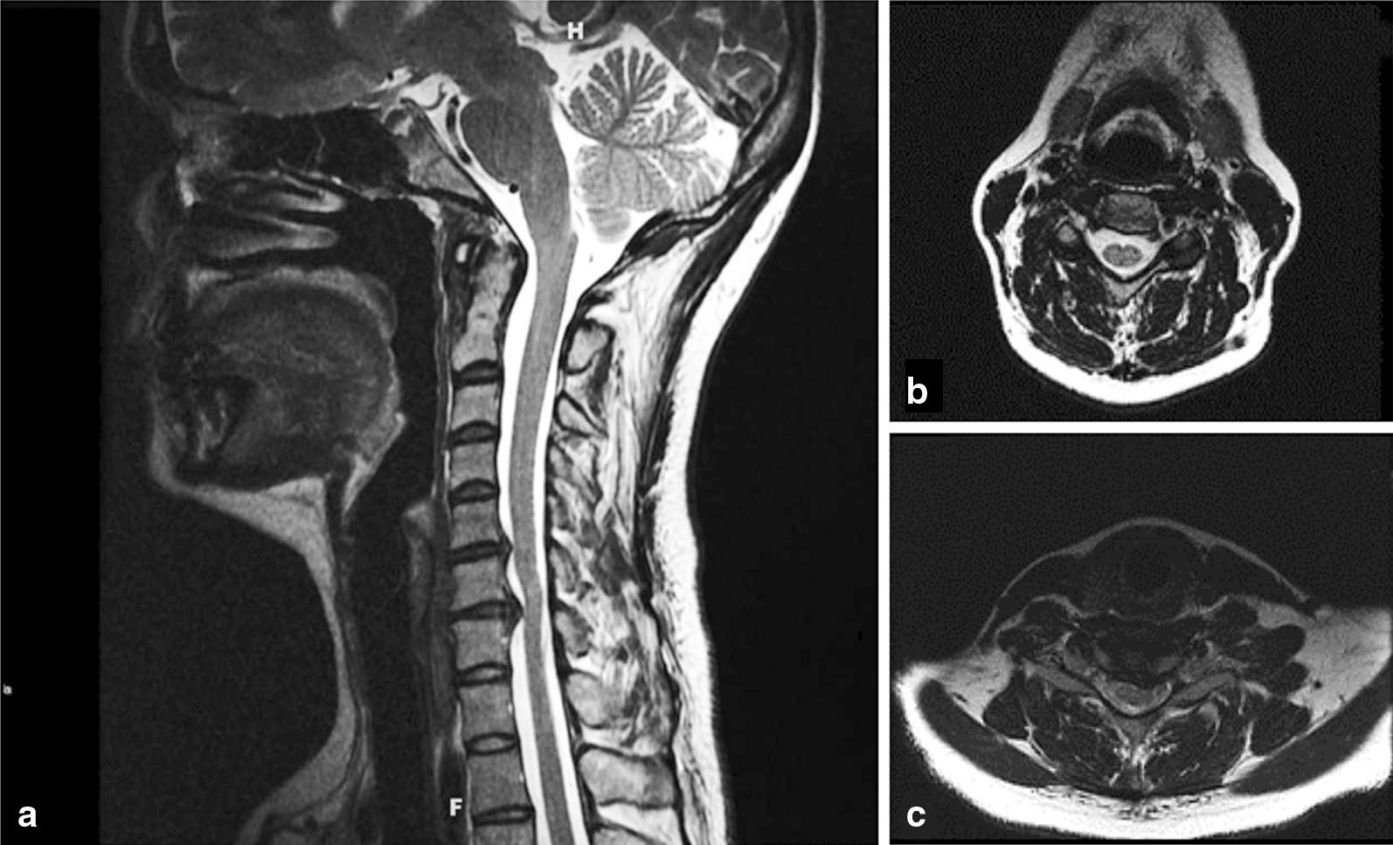
Cervical spine MRI images (Patient 2). **a** The sagittal cervical cord MRI image showed a clivo-axial angle of 124°, as well as a disc protrusion at C5–6 narrowing the cervical canal to 9 mm and a left paracentral disc protrusion at C6–7 narrowing the AP sagittal canal diameter to 7 mm, with indentation of the left ventral cord surface and myelomalacia. **b** A transverse image at C5–6. **c** A transverse image at C6–7 illustrating cord indentation

By age 35, she had daily profound fatigue, generalized weakness, lightheadedness, and presyncopal episodes. She was unable to work, and required a wheelchair for trips outside the home. Any trips out of the house precipitated up to 2 weeks of exacerbation of symptoms. The presence of generalized 3+ hyper-reflexia prompted a repeat MRI of the cervical spine that showed a 9 mm cervical canal at C5–6 and a 7 mm cervical canal diameter at C6–7 (Fig. 2). Her spine surgeon identified an absent gag reflex, diminished strength in triceps and wrist flexors, and 4+ DTRs at the ankles. The Hoffman sign was negative.

At age 35, she underwent a hybrid anterior cervical disc fusion and disc replacement. She noted a general sense of improvement in the first week after surgery. She was able to walk a 1/2 mile several days in a row and no longer needed a wheelchair for doctor visits. Activity tolerance increased. The burning in both upper and lower limbs with standing resolved at 6 months, and she was symptom free during 5 min of standing. By 8 months after surgery she was able to walk for 35 min a day with less relapse of symptoms afterwards, and could remain upright and active for most of the day. At 20 months, she could exercise on an elliptical machine and recumbent bicycle, perform daily house-keeping chores, run multiple errands in a day, and paint the interior rooms of her parents’ house. She was able to successfully discontinue 3 of the 4 antidepressant/anti-anxiety medications.

*Patient 3* was previously healthy, working full-time until age 31, when she developed profound fatigue upon return from an overseas trip. A tilt test was performed to investigate orthostatic intolerance as the possible etiology of her symptoms, during which she became syncopal and unresponsive at 25 min into the test, with a junctional bradycardia and an undetectable blood pressure, consistent with neurally mediated syncope. Her blood pressure and HR normalized with return to supine position. She stopped working after 4 months due to persistent fatigue. She also reported unrefreshing sleep, post-exertional malaise, difficulty with concentration, headache, arthralgias and myalgias, nausea, lightheadedness, tremulousness, visual disturbances, and excessive thirst. After 6 months of symptoms, she met the clinical criteria for ME/CFS. She reported decreased fine motor coordination. Anxiety and depression emerged as the other symptoms progressed.

After 2 years of symptoms, a 10-min standing test demonstrated a HR change of 34 bpm consistent with POTS. Her physical exam was notable for joint hypermobility (Beighton score 5). On upper limb neurodynamic testing with a median nerve bias, elbow extension was limited by 46 and 22° on the left and right, respectively. Neurological findings included brisk DTRs in the 2–3+ range with spread of the patellar reflex, a fine resting tremor, and a positive Hoffman sign bilaterally. Treatments for her orthostatic intolerance were ineffective or poorly tolerated. Progression of symptoms led to an inability to work, drive, or walk more than short distances. She required wheelchair use for most trips outside the house.

**Fig. 3:**
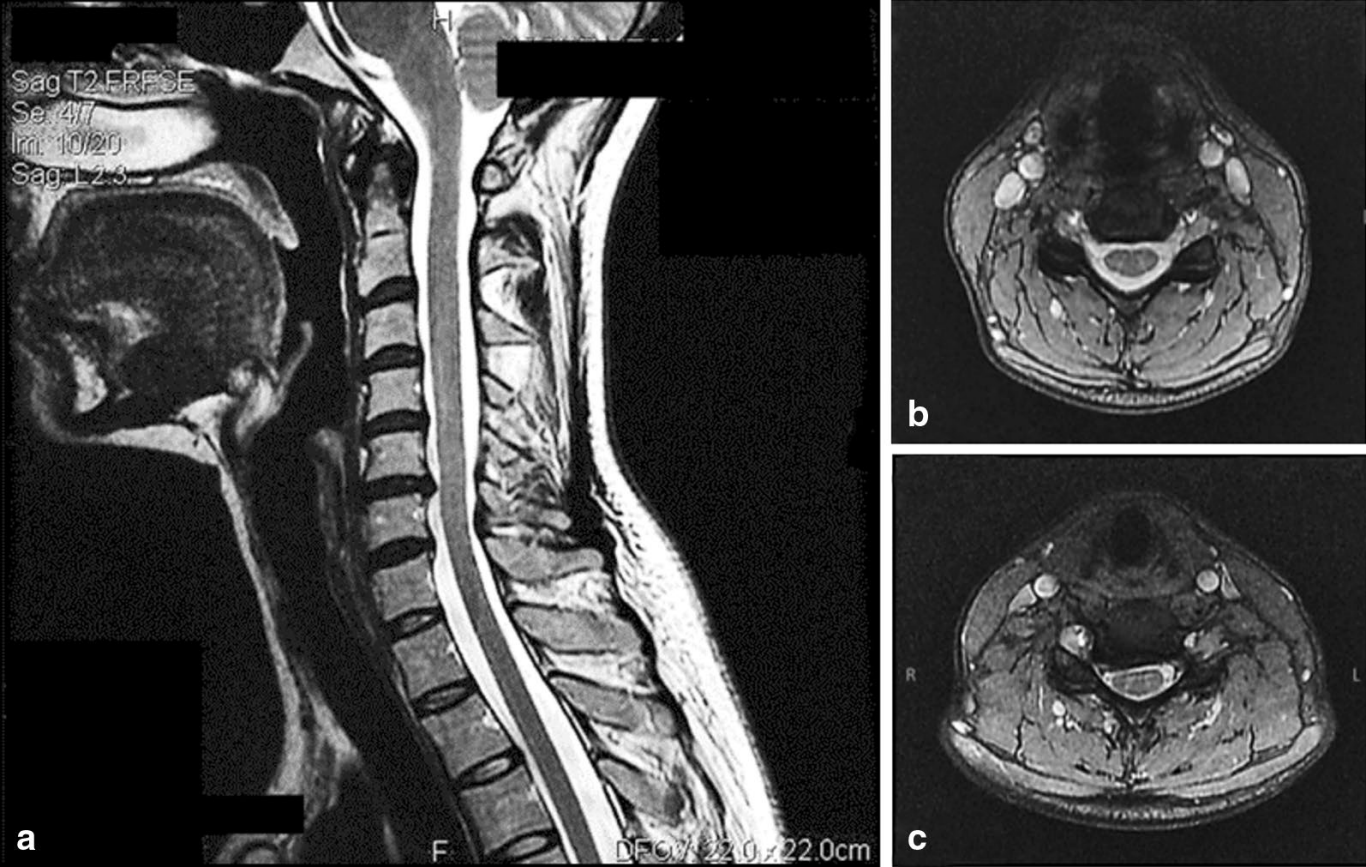
Cervical spine MRI images (Patient 3). **a** The sagittal cervical cord MRI showed disc protrusion with right ventral cord contact at C5–6, and an AP canal diameter of 8.5 mm at that segment. **b** A transverse image at C4–5. **c** A transverse image at C5–6 illustrating cord indentation

Her cervical spine MRI at age 35 showed spondylosis at C5–6, with a canal diameter of 8.5 mm (Fig. 3). Her spine surgeon noted right wrist weakness and some loss of sensation in the median nerve distribution. She elected to undergo anterior C5–6 disc replacement surgery. Tremor, headache, and right shoulder and neck discomfort all resolved in the first week after surgery, at which time she informally noted a lower resting HR and an increase in activity tolerance. She was able to build strength and endurance through gradual increases in exercise, and tolerated 45 min of symptom-free standing. By 3 months post-operatively she was able to complete 5 downhill ski runs. At 7 months post-operatively she was able to engage in regular vigorous physical exercise without provoking increased malaise. She tolerated a reduction in her venlafaxine dose. One year after surgery she reported minimal physical limitations. Cognition improved and she was able to resume reading complex literature.

**Fig. 4:**
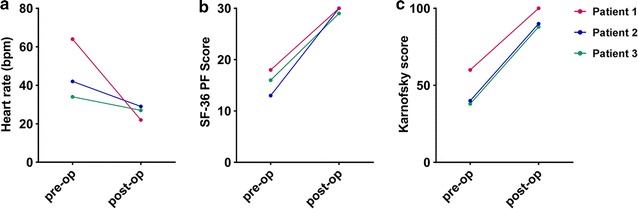
**a** Improvement in the maximum increase in heart rate from supine to the peak value during 10-min of standing, pre- and post-operatively. The standing tests were performed at varying intervals after surgery (after 4 years for Patient 1, and after 1 year for Patients 2 and 3). All three patients had resolution of POTS. **b** Self-reported scores of the physical function subscale of the SF-36 measure, pre- and post-operatively at the same post-operative intervals as in a. **c** Improvements on the physician-assigned Karnofsky scores for each patient, pre- and 1-year post-operatively

Figure 4 illustrates the improvements in the heart rate changes during standing tests, as well as the Karnofsky and SF-36 scores for the 3 patients.

## Discussion

This case series describes three young women with moderately severe ME/CFS and one- or two-level cervical canal stenosis. Their narrowest AP cervical canal diameters were between 6 and 8.5 mm. Surgical management was associated with a marked improvement in myelopathic symptoms, resolution of lightheadedness and hemodynamic dysfunction, improvement in activity levels, and improvement in global ME/CFS symptoms. In the absence of evidence from randomized trials, we cannot exclude a coincidental improvement or a placebo response to surgery. However, we consider this less likely given the myelopathic examination findings, the demonstration of spinal cord compression on MRI scans, and the lack of a placebo response to multiple previous interventions that included cognitive behavioral and psychiatric treatment. The prompt post-surgical restoration of more normal function instead suggests that the spinal cord compression had a causal role in symptoms, and emphasizes the importance of a careful search for myelopathic examination findings in those with ME/CFS, especially when individuals with severe impairment are not responding to treatment.

The diagnosis of cervical stenosis in all three patients was delayed for several years. In patient 1, who had cervical spondylosis superimposed on congenital cervical stenosis, an overlooked historical clue to her diagnosis was the history of congenital cervical stenosis in her mother. Patient 1′s deep tendon reflexes were never brisk, and her Hoffman sign was only positive after several years of normal neurological exam findings. The Hoffman sign is present in a variable proportion of those with cervical myelopathy, appearing earlier in myelopathy than the Babinski sign [26]. While it can be detected in healthy individuals, among 16 adults with positive Hoffman signs and no cervical symptoms, 15 (94%) had evidence of cord compression on MRI [27]. It is possible that the Hoffman sign would have been more consistently abnormal in patient 1 if it had been elicited after multiple flexion and extension movements of the neck as described by Dennow and Meadows [28].

The other two patients had consistently pathological neurological examination findings. Both had brisk deep tendon reflexes, one with ankle clonus and the other with a positive Hoffman sign. Both individuals had been evaluated by more than one experienced neurologist, and the reflex findings had been attributed to non-pathological or psychosomatic causes. We speculate that a negative bias toward the ME/CFS label and the presence of co-morbid anxiety may have distracted attention from the neurological findings. Of interest, all 3 patients reported less anxiety in daily life after surgery, and all were able to reduce or eliminate their anti-anxiety medication. A reduction in anxiety following cervical decompression has been reported elsewhere [29].

Our findings are consistent with the observations of Heffez and colleagues in adults with fibromyalgia [30], a condition with a substantial symptom overlap with ME/CFS [31]. Among 270 adults referred to evaluate whether there was a neurological basis for their fibromyalgia symptoms (none of whom had previously been diagnosed with a cervical myelopathy), myelopathic symptoms included neck/back pain (96%), instability of gait (86%), subjective grip weakness (83%), paresthesias (80%), dizziness (71%), and numbness of the hands and feet (69%). Hyper-reflexia was identified in 64%, a positive Romberg sign in 28%, a positive Hoffman sign in 26%, ankle clonus in 25%, and impaired tandem walk in 23%. The AP spinal canal diameter at the level of the C5/6 intervertebral disc measured 10 mm or less in 46% of participants [30]. A subset of this cohort underwent treatment of cervical myelopathy by surgical (N = 40) or non-surgical (N = 31) means [29]. The surgical group was not restricted to those with cervical spine stenosis alone, as an unreported number underwent posterior fossa decompression for Chiari malformation. There were no baseline differences in symptom prevalence at baseline, and neurologic signs were similar between the surgical and non-surgical patients. As expected, the patients in the surgical group had a narrower mid-sagittal spinal canal diameter at C5/6 and C6/7. At the 1-year follow-up, those in the surgical group experienced a significant improvement in all symptoms attributed to fibromyalgia (including fatigue, impaired concentration, and body pain), significantly higher quality of life scores on the SF-36 physical and mental health subscales, and significant improvement in depression and anxiety scores. Holman has also reported intermittent cervical cord compression with flexion and extension MRI studies in 71% of fibromyalgia patients [32]. While referral bias in those studies does not allow an estimate of the prevalence of cervical myelopathy in unselected fibromyalgia patients, these studies confirm that a subset of individuals with fibromyalgia, many of whom also meet criteria for ME/CFS [31], have potentially treatable cervical myelopathy or dynamic cervical cord compression. Our series extends these observations to those whose symptoms are dominated more by profound fatigue, post-exertional worsening of symptoms, and autonomic dysfunction, and less by widespread pain.

Of interest, all three patients in our series had potentially pathological clivo-axial angles. The decision was made to operate on the bulging cervical discs as a first step, as it was the least invasive surgical intervention. After 32–70 months of follow-up there has been no need for further surgery. We are unsure whether the combined presence of abnormal clivo-axial angle and cervical canal stenosis poses a greater risk of neurological symptoms, but this possibility should be kept in mind in future investigations, especially in light of the observation than an abnormal clivo-axial angle is capable of causing deformative stress within the spinal cord [20].

Our paper also draws attention to the potential for neurally mediated syncope and POTS to be associated with cervical myelopathy. Both hemodynamic abnormalities are strongly associated with ME/CFS [1, 33, 34], and treatment of the orthostatic intolerance can lead to improvements in ME/CFS symptoms and function [33]. One limitation of this series is that the standing tests were performed at varying intervals after surgery. Given the positive impact of exercise on improving orthostatic tolerance in those with POTS [35], we cannot be certain that the improvements in heart rate response during standing were related primarily to the cervical decompression or to the advancement of exercise. However, all three patients reported improvements in lightheadedness and tolerance of upright posture with the first week of surgery, suggesting that the improvement in circulatory symptoms preceded and likely enabled the improvement in activity.

The global improvements noted following surgical decompression in these three patients would suggest a neurological basis of many of their ME/CFS symptoms. Recent research has proposed that ME/CFS symptoms might be related to central nervous system inflammation, central sensitivity, or reduced cerebral blood flow [36–38]. The mechanism by which cervical cord compression could influence these putative mechanisms of ME/CFS symptom generation is unclear, but other work has shown that application of a longitudinal neural strain using a passive straight leg raise can increase the intensity of fatigue, pain, headaches, cognitive dysfunction, and lightheadedness in those with ME/CFS [39]. These findings would be relevant given the increased biomechanical strain within the cord that can occur with cervical spondylosis [14, 15]. Subclinical autonomic nervous system dysfunction has been described among those with compressive cervical myelopathy [40]. Because autonomic fibers also traverse through the spinal cord, Srihari and colleagues have proposed that autonomic involvement in compressive cervical myelopathy should be expected. They hypothesize that cervical decompression can lead to improvement in symptoms by reducing cord edema, improving venous drainage, and improving cord perfusion [40]. Hoshimaru has hypothesized that improvements in neuropsychological function among patients with cervical spondylotic myelopathy after decompression surgery could be related to improvement in cerebrospinal flow within the spinal canal, which in turn would be expected to lead to improvement in cerebral blood flow [41]. These mechanisms would also be potential contributors to changes in ME/CFS and autonomic symptoms.

This case series emphasizes an overlap in certain symptoms of ME/CFS and myelopathy. The extent of that overlap will require more attention to the systemic symptoms of those with traditional cervical myelopathy (who do not have a diagnosis of ME/CFS), and to the myelopathic symptoms and exam findings of those with ME/CFS. Although cervical stenosis does not appear to be a common abnormality among those with ME/CFS, its prevalence has not been the subject of formal study. We would expect cervical stenosis to be more common among those with more severe forms of the illness. The presence of myelopathic symptoms and signs warrants further radiologic investigation, after which care will need to be taken to define the optimal candidates for surgical decompression. This case series establishes the potential for spondylotic cervical myelopathy to contribute in an important way to symptoms in a subset of those with moderate to severe ME/CFS and orthostatic intolerance. It will be worthwhile to investigate these observations further in larger prospective studies. To evaluate more definitively whether cervical spinal stenosis can cause ME/CFS symptoms will require a randomized trial of surgery in individuals with ME/CFS who are found on further investigation to have cervical spinal stenosis and classical indications for cervical decompression surgery.

## Conclusions

This case series reports three young women with ME/CFS and orthostatic symptoms that were refractory to medical and psychiatric management. All were ultimately found to have cervical spinal stenosis. The prompt post-surgical restoration of more normal function suggests that cervical spine stenosis contributed to the pathogenesis of their ME/CFS symptoms. The improvements following surgery emphasize the importance of a careful search for myelopathic examination findings in those with ME/CFS, especially when individuals with severe impairment are not responding to treatment.

## Abbreviations

ME/CFS: myalgic encephalomyelitis/chronic fatigue syndrome
POTS: postural tachycardia syndrome
MRI: magnetic resonance imaging
SF-36: Medical Outcomes Study 36-item short-form health survey
PF: physical function
HR: heart rate
Bpm: beats per minute
DTRs: deep tendon reflexes
AP: antero-posterior
